# Expression Profile of Cytokines and Enzymes mRNA in Blood Leukocytes of Dogs with Leptospirosis and Its Associated Pulmonary Hemorrhage Syndrome

**DOI:** 10.1371/journal.pone.0148029

**Published:** 2016-01-29

**Authors:** Carla A. Maissen-Villiger, Ariane Schweighauser, H. Anette van Dorland, Claudine Morel, Rupert M. Bruckmaier, Andreas Zurbriggen, Thierry Francey

**Affiliations:** 1 Division of Small Animal Internal Medicine, Department of Clinical Veterinary Medicine, Vetsuisse Faculty University of Bern, Bern, Switzerland; 2 Division of Veterinary Physiology, Department of Clinical Research and Veterinary Public Health, Vetsuisse Faculty University of Bern, Bern, Switzerland; 3 Division of Experimental Clinical Research, Department of Clinical Research and Veterinary Public Health, Vetsuisse Faculty University of Bern, Bern, Switzerland; University of São Paulo School of Medicine, BRAZIL

## Abstract

**Background:**

Dogs with leptospirosis show similar organ manifestations and disease course as human patients, including acute kidney injury and pulmonary hemorrhage, making this naturally-occurring infection a good animal model for human leptospirosis. Expression patterns of cytokines and enzymes have been correlated with disease manifestations and clinical outcome in humans and animals. The aim of this study was to describe mRNA expression of pro- and anti-inflammatory mediators in canine leptospirosis and to compare it with other renal diseases to identify patterns characterizing the disease and especially its pulmonary form.

**Methodology and Principal Findings:**

The mRNA abundance of cytokines (IL-1α, IL-1β, IL-8, IL-10, TNF-α, TGF-β) and enzymes (5-LO, iNOS) was measured prospectively in blood leukocytes from 34 dogs with severe leptospirosis and acute kidney injury, including 22 dogs with leptospirosis-associated pulmonary hemorrhages. Dogs with leptospirosis were compared to 14 dogs with acute kidney injury of other origin than leptospirosis, 8 dogs with chronic kidney disease, and 10 healthy control dogs. Canine leptospirosis was characterized by high 5-LO and low TNF-α expression compared to other causes of acute kidney injury, although the decreased TNF-α expression was also seen in chronic kidney disease. Leptospirosis-associated pulmonary hemorrhage was not characterized by a specific pattern, with only mild changes noted, including increased IL-10 and decreased 5-LO expression on some days in affected dogs. Fatal outcome from pulmonary hemorrhages was associated with low TNF-α, high IL-1β, and high iNOS expression, a pattern possibly expressed also in dogs with other forms of acute kidney injury.

**Conclusion:**

The patterns of cytokine and enzyme expression observed in the present study indicate a complex pro- and anti-inflammatory response to the infection with leptospires. The recognition of these signatures may be of diagnostic and prognostic relevance for affected individuals and they may indicate options for newer therapies targeting the identified pathways.

## Introduction

Leptospirosis is a widespread bacterial zoonosis affecting over 150 different species of mammals worldwide [1,2]. It is an emerging infectious disease for humans and dogs in many countries and is caused by a strictly aerobic gram-negative spirochete of the genus *Leptospira* [3]. A wide range of domestic and wild animals, in particular rodents, are chronic, asymptomatic carriers of leptospires and shed the pathogenic spirochetes in their urine. Infection of dogs and humans occurs directly through contact with urine of carriers or indirectly through intake of contaminated soil or water [1,2].

Dogs suffering from leptospirosis show similar clinical manifestations and disease course as affected human patients, including acute kidney injury (AKI), liver failure, systemic hemorrhagic syndrome, and pulmonary hemorrhages, known as Weil’s syndrome. As in humans, the syndrome of leptospirosis-associated pulmonary hemorrhage (LAPH) has been reported recently in European dogs and represents a severe manifestation with high morbidity and letality rates, reaching 70–77% and 44–48%, respectively [4,5,6,7,8]. The pathophysiological mechanism underlying this massive pulmonary erythrocyte extravasation with alveolar flooding remains however elusive [9,10]. A direct effect of an unidentified bacterial toxin on pulmonary microvasculature or an indirect effect mediated by the host immune response are the two mechanisms most often considered [11].

The expression of cytokine and enzyme mRNA has been investigated previously in human and non-canine animal models of leptospirosis [12,13,14]. Secreted by cells of the immune system, they lead to activation, differentiation, and proliferation of their target cells and may therefore serve as diagnostic and prognostic markers or as indicators of pathophysiological mechanisms involved in various aspects of a disease [15,16,17]. A study performed on hamsters showed up-regulated gene expression of both pro- and anti-inflammatory cytokines with fatal outcome in a LD50 model of leptospirosis, suggesting that cytokine expression could be a predictor of outcome in leptospirosis [18]. Recent research indicates a potential role of different cytokines and enzymes on the outcome and severity of the disease, including development of bleeding as a response to the sepsis-like manifestations in humans and hamsters [6,14].

No data are currently available on the expression of cytokines and inflammatory enzymes in canine leptospirosis, in particular for its pulmonary form. However, a high prevalence of clinically well-characterized dogs with severe leptospirosis requiring hospitalization and advanced therapy similar to the human disease makes this naturally-occurring infection a good model to study the pathophysiological mechanisms underlying human and animal LAPH [5,9,19]. This model offers the opportunity to research cytokine and enzyme expression under clinical, non-laboratory, conditions and thus to evaluate further methods to improve prevention and therapy of this severe form of disease associated with high mortality in both humans and canines.

The aims of this study were thus to identify patterns of cytokine or enzyme expression in blood leukocytes characteristic of canine leptospirosis, indicating the presence of LAPH, or associated with outcome. Our main hypotheses were that certain cytokines and enzymes are differently expressed in dogs with leptospirosis compared to kidney diseases of other origin (AKI not due to leptospirosis (AKInL), chronic kidney disease (CKD)), in dogs with LAPH compared to dogs without LAPH (nLAPH), and in dogs surviving leptospirosis and LAPH compared to dogs with fatal outcome. For this, we evaluated the mRNA expression of a panel of immune related cytokines (IL-1α, IL-1β, IL-8, IL-10, TNF-α, TGF-β) and enzymes (5-LO, iNOS) in blood leukocytes from dogs with AKI and leptospirosis, including dogs with and without LAPH. Affected dogs were compared to dogs with AKInL, to dogs with CKD, and to healthy dogs.

## Materials and Methods

### Ethics Statement

All experimental procedures used in this study followed the Swiss Law on Animal Protection and were approved by the Ethical Research Committee of the Small Animal Clinic of the Vetsuisse Faculty University of Bern, Switzerland and by the Committee of Animal Experiments of the Canton Bern, Switzerland. Written informed consent was obtained from all dog owners.

### Animals and Group Definitions

Dogs affected with AKI or CKD were recruited among the patients evaluated and treated by the Nephrology Service of the Small Animal Clinic of the Vetsuisse Faculty University of Bern, Switzerland over a 12 months period. Clinically healthy dogs belonged to the staff of the Small Animal Clinic and to volunteering owners.

The dogs were divided into the following 4 main disease groups: dogs with leptospirosis (LEPTO), dogs with AKI from other causes than leptospirosis (AKInL), dogs with chronic kidney disease (CKD), and healthy control dogs (HC). The LEPTO group was further divided into the subgroups of dogs with leptospirosis-associated pulmonary hemorrhage (LAPH) and dogs without LAPH (nLAPH).

A case of leptospirosis was defined based on consistent clinical presentation, including dogs with renal, hepatic, pulmonary or hemorrhagic manifestations, with laboratory confirmation [20]. The latter could be obtained with either paired microscopic agglutination test (MAT) serology; single sample MAT serology; or PCR on blood, urine, kidney or liver tissue [1,20]. Serology with MAT was performed as previously described by the accredited National Reference Laboratory for Leptospirosis (Institute of Veterinary Bacteriology, National Center for Zoonoses, Bacterial Animal Diseases and Antimicrobial Resistance, Vetsuisse Faculty, University of Bern, Switzerland) according to the Guidelines of the World Health Organization International Leptospirosis Society [5,21]. Briefly, the MAT was performed using a panel of ubiquitous and locally prevalent serovars, including *L*. *interrogans* serovars Australis, Autumnalis, Bataviae, Bratislava, Canicola, Hardjo, Icterohaemorrhagiae, Pomona, Sejroe, and Tarassovi and *L*. *kirschneri* serovar Grippotyphosa. Sera were initially screened at a dilution of 1:100. Samples with a positive reaction were titrated in a serial two-fold dilution to a maximum of 1:3200 and the end-point titer was recorded. For case definition, paired serology with fourfold rise in sequential titers at a 1–3 week interval was considered first-choice. For animals where a second sample could not be obtained (typically due to early death), a single sample obtained at presentation with a titer ≥1:800 was considered positive, as previously demonstrated [19,20].

Acute kidney injury was defined as an acute onset of renal azotemia and compatible historical, clinical, clinicopathological and ultrasonographic findings [22]. Only dogs with an identified cause of AKI other than leptospirosis or with negative paired MAT serology were included in the group of AKInL. Dogs with evidence of underlying CKD were excluded from this groups, even when the clinic was dominated by the acute injury.

The LEPTO group was divided into two subgroups according to the presence or the absence of pulmonary hemorrhage using radiographic imaging of the thorax [23]. Thoracic radiographs were performed in all dogs with AKI at initial presentation, independently of clinical manifestations. They were repeated subsequently only in dogs with clinical signs of respiratory disease. Animals with radiographic evidence of LAPH were included into the LAPH group. Dogs that did not show evidence of pulmonary bleeding at any point during hospitalization were included into the nLAPH group.

Chronic kidney disease was diagnosed based on history, physical examination, blood pressure, complete blood count, chemistry profile, urinalysis, urinary protein to creatinine ratio, and abdominal ultrasound. Dogs with acute decompensation of CKD were excluded. Healthy control dogs were confirmed healthy based on history, physical examination, blood pressure, complete blood count, chemistry profile, urinalysis, and negative MAT for leptospirosis.

Exclusion criteria for all groups included any treatment with non-steroidal anti-inflammatory drugs or corticosteroids within one week of sampling or any suspicion or evidence of preexisting pulmonary disease.

Treatment and clinical or laboratory re-evaluation of dogs hospitalized for AKI (LEPTO and AKInL) were assessed and decided individually, based on clinical needs. All evaluations and treatment decisions were taken by the 2 clinical nephrologists (AS, TF). During the whole hospitalization, the dogs were maintained under continuous monitoring in the intensive care unit, including surveillance of their vital parameters and pain assessment 4 times daily. According to standard therapy protocols for dogs with AKI, all dogs were treated for at least 4–7 days with opioids (buprenorphine or fentanyl) to minimize pain associated with their disease. In cases of pain non-responsive to standard use of analgesics, a consultation was obtained with a specialized anesthetist for recommendations on the use of more advanced analgesic protocols. Since the included dogs were client-owned patients of the teaching hospital, final decision concerning survival endpoints were in the hand of their owners. However, humane euthanasia was strongly recommended for animals suffering severe dyspnea non-responsive to oxygen therapy or pain and distress non-responsive to the use of advanced analgesic protocols. When a decision of euthanasia was taken by the dog owners, this was performed by intravenous injection of butorphanol and propofol, followed by an overdose of pentobarbital. Outcome data are reported as global outcome since a decision of euthanasia is commonly multifactorial and not guided solely by clinical parameters. No unexpected death was observed in the study dogs.

### Blood Sampling

Whole blood (500 μl) was collected from the cephalic, saphenous or jugular vein and immediately added to the RNAprotect^®^ Animal Blood Tube (Qiagen, Switzerland) to stabilize RNA. After gently mixing the tube for 8–10 times, the sample was stored upright at 15–25°C for 24 hours, then at 4°C for 24 hours, and finally at -20° to -80°C until analyzed.

Blood sampling took place together with the regular blood rechecks used for clinical monitoring on seven consecutive days after admission or until day of death for groups LEPTO and AKInL. Three samples were collected within 10 days at the time of regular rechecks for evaluation of disease stability in dogs of the CKD group. Healthy control dogs (HC group) were sampled daily for 5 consecutive days to assess stability of cytokine expression and non-disease factors. Sampling was performed between 8 to 10 a.m. whenever possible. The time for the first blood collection was however dependent on the time of presentation to the clinic for each dog.

### Total RNA Extraction and cDNA Synthesis

Samples kept at -80°C were thawed overnight at 5°C for 12 hours and then stored at room temperature for 2 hours before starting the procedure. Total RNA extraction was performed with the RNeasy^®^ Protect Animal Blood Kit (Qiagen, Switzerland) according to the manufacturer’s protocol. To ensure maximum elution efficiency the protocol was slightly modified (step 17, RNA elution) as follows: 20 μl of the elution buffer was pipetted directly onto the RNeasy^®^ MinElute spin column membrane, the lid gently closed and incubated on the bench top for 2 minutes. The spin column was then centrifuged for 1 min at 8000 x g. This step was repeated with 10 μl of the elution buffer. Quantity and purity of the extracted RNA were determined by absorbance at 260 and 280 nm using the NanoDrop ND-2000 spectrophotometer (NanoDrop Technologies Inc., Wilmington, DE). The RNA eluate was stored at -80°C if not used immediately.

The extracted total RNA was reverse transcribed with the SuperScript^®^ VILO™ cDNA Synthesis Kit (Invitrogen Corporation, Switzerland). 5X VILO™ Reaction Mix + 10X SuperScript^®^ Enzyme Mix were mixed as described by the manufacturer to obtain a Master Mix. Total RNA (28 μl, 0.2 μg) of every sample was put into a 0.2 μl tube and 12 μl of the Master Mix was added. Tube contents were gently mixed before putting the tubes into the Mastercycler. Tubes were first incubated at 25°C for 10 minutes, then at 42°C for 120 minutes. Finally the reaction was terminated at 85°C for 5 minutes and the obtained cDNA was stored at -20°C until polymerase chain reaction was performed.

### Primers and Primer Design (Table 1)

The primers for the housekeeping gene ubiquitin, for IL-1β, TNF-α, 5-LO, IL-10 and TGF-β were chosen based on previous publications [24,25,26]. For IL-1α, IL-8 and iNOS, corresponding primers were designed applying the following criteria: product size between 150 and 400 base pairs, primer length about 20 base pairs, ratio GC to TA more or less equal, end of primer preferably G or C, intron spanning if possible. Sequences of *Canis lupus familiaris* IL-1α, IL-8 and iNOS were available from the National Center for Biotechnology Information (http://www.ncbi.nlm.nih.gov/nuccore). Designed primers were tested with 2.5% agarose gel electrophoresis followed by staining with ethidium bromide. As a marker a DNA step ladder (Promega, Switzerland) was used.

**Table 1:** Detailed primers and conditions used for real-time PCR assays.

Gene product^1^		Primer sequence 5'-3'	GeneBank accession no.	Annealing temperature (C°)	Amplicon length (bp)
**IL-1α**	For	GATGGCCAAAGTTCCTGACC	NM_001003157.2	60	230
	Rev	GAATCTTCCCATTGGCTGCC			
**IL-1β**	For	CCCTGGAAATGTGAAGTGCT	NM_001037971.1	60	242
	Rev	TATCCGCATCTGTTTTGCAG			
**IL-8**	For	GCTGAGAAACAAGATCCGTG	NM_001003200.1	60	343
	Rev	CTGTAGGTGAGGTGGAAAGA			
**IL-10**	For	CTCCCTGGGAGAGAAGCTCAA	U33843	60	72
	Rev	ACAGGGAAGAAATCGGTGACA			
**TNF-α**	For	TCATCTTCTCGAACCCCAAG	EU249361.1	60	157
	Rev	ACCCATCTGACGGCACTATC			
**TGF-β**	For	CAAGTAGACATTAACGGGTTCAGTTC	L34956	60	70
	Rev	GGTCGGTTCATGCCATGAAT			
**5-LO**	For	GTGGACACGTGCAGATGGTG	NM_001205113.1	60	166
	Rev	GTGAACGTCTTGATGGCCTC			
**iNOS**	For	AGCGCTACAACATCCTGGAG	AF077821.1	60	270
	Rev	GGAACACAGGAGTGATGCTC			
**UBQ**	For	CAGCTAGAAGATGGCCGAAC	AB032025	60	199
	Rev	ACTTCTTCTTGCGGCAGTTG			

^1^ IL-1α, interleukin-1alpha; IL-1β, interleukin-1beta; IL-8, interleukin-8; IL-10, interleukin-10; TNF-α, tumor necrosis factor alpha; TGF-β, transforming growth factor beta; 5-LO, 5-lipoxygenase; iNOS, inducible nitric oxide synthase; UBQ, ubiquitin.

#### Real-Time PCR

PCR quantification was performed with the Rotor-Gene™ 6000 (Corbett Research, Sydney, Australia), using the software version 1.7.40. Fluorescence take off was calculated with the “threshold” program option. The following three-step PCR program was used: denaturation for 10 min at 95°C, 40 cycles of amplification (each consisting of 15 s at 95°C, primer-specific annealing temperature for 30 s, extension at 72°C for 20 s, and quantification of fluorescence), and finally a melting curve program (60–95°C). The mRNA levels were calculated relatively to the expression of the housekeeping gene ubiquitin, which was stable across time-points.

### Statistical Analysis

Variance analysis using the PROC GLM procedure of SAS (2001) was performed for each observed parameter with disease group as fixed effect (Tables 2, 3 and 4). Because of early death of some dogs with AKI, the analysis was performed separately for survivors and non-survivors. Data are reported as means ± standard error of the mean (SEM) and were considered significant if *P* <0.05 or a trend if *P* <0.10. In Table 3, mRNA abundance data are presented as the area under the curve for day 1 to 3. This time frame was chosen based on initial observations as the most representative for the underlying disease before treatment effects and iatrogenic complications potentially affected cytokine and enzyme expression. One dog of the CKD group had to be excluded from the calculation of the area under the curve for Table 3, because only two instead of three samples could be obtained.

**Table 2:** Mean ± SEM of clinical characteristics of all dogs on the day of or one day after admission to the clinic.

	Disease group^1^							ANOVA (*P*-value)			
	LEPTO										
	LAPH	nLAPH	AKInL	CKD	HC	LAPH	LEPTO	LEPTO	LEPTO	AKInL	CKD
Variable^2^	(n = 22)	(n = 12)	(n = 14)	(n = 8)	(n = 10)	vs nLAPH	vs AKInL	vs CKD	vs HC	vs HC	vs HC
***Blood parameters***											
PCV (l/l)	0.30 ± 0.01	0.36 ± 0.03	0.36 ± 0.02	0.23 ± 0.03	0.44 ± 0.01	0.03	0.06	< 0.01	< 0.001	<0.01	<0.001
Tc (10^9^/l)	173 ± 23.3	182 ± 37.1	175 ± 30.6	198 ± 36.8	243 ± 15.6	0.83	0.99	0.61	0.07	0.09	0.24
Lc (10^9^/l)	19.0 ± 2.18	17.0 ± 1.76	11.8 ± 1.56	9.82 ± 1.38	9.32 ± 0.94	0.55	0.01	0.01	< 0.01	0.24	0.76
K (mmol/l)	5.21 ± 0.36	3.47 ± 0.17	5.11 ± 0.45	4.16 ± 0.17	3.99 ± 0.06	< 0.01	0.34	0.47	0.25	0.05	0.31
P (mmol/l)	4.74 ± 0.43	3.35 ± 0.39	4.93 ± 0.65	4.76 ± 0.61	1.28 ± 0.10	0.04	0.30	0.49	< 0.001	<0.001	<0.001
Urea (mmol/l)	62.3 ± 5.39	44.8 ± 6.29	62.1 ± 7.43	71.8 ± 7.65	6.24 ± 0.49	0.05	0.48	0.11	< 0.001	<0.001	<0.001
Crea (μmol/l)	836 ± 88.3	561 ± 95.9	912 ± 104	920 ± 124	81.4 ± 3.85	0.06	0.18	0.25	< 0.001	<0.001	<0.001
***Clinical Parameters***											
PR (beats/min)	110 ± 6.59	101 ± 8.90	92.1 ± 6.53	114 ± 9.28	81.2 ± 7.38	0.38	0.12	0.56	0.02	0.29	0.01
RR (breaths/min)	37.9 ± 5.56	21.2 ± 3.06	23.6 ± 2.96	25.1 ± 1.68	15.0 ± 3.95	0.04	0.20	0.45	0.03	0.09	0.06
T (°C)	37.6 ± 0.16	37.8 ± 0.24	37.3 ± 0.13	37.7 ± 0.17	38.3 ± 0.12	0.66	0.11	0.96	0.02	<0.001	<0.01

^1^ LAPH, dogs with leptospirosis and associated pulmonary hemorrhage and acute kidney injury; nLAPH, dogs with leptospirosis, but without associated pulmonary hemorrhage and with acute kidney injury; AKInL, dogs with acute kidney injury of other origin than leptospirosis (leptospirosis-negative dogs); CKD, dogs with chronic kidney disease; HC, healthy control dogs; LEPTO, dogs with leptospirosis (LAPH+nLAPH).

^2^ PCV, packed cell volume; Tc, platelets; Lc, leukocytes; K, potassium; P, phosphorus; Crea, creatinine; PR, pulse rate; RR, respiratory rate; T, temperature

**Table 3:** Mean ± SEM area under the curve of relative mRNA expression (CT, log_2_) of genes encoding cytokines and enzymes in whole blood of diseased dogs during the first three days after admission to the clinic (non-survivors excluded).

	Disease group^1^					ANOVA (*P*-value)					
	LEPTO										
	LAPH	nLAPH	AKInL	CKD	HC	LAPH	LEPTO	LEPTO	LEPTO	AKInL	CKD
Variable^2^	(n = 13)	(n = 11)	(n = 10)	(n = 7)	(n = 10)	vs nLAPH	vs AKInL	vs CKD	vs HC	vs HC	vs HC
***Cytokines***											
IL-1α	12.2 ± 0.34	12.4 ± 0.30	12.6 ± 0.43	13.1 ± 0.41	10.2 ± 0.24	0.57	0.52	0.09	< 0.001	<0.001	<0.001
IL-1β	14.2 ± 0.43	14.7 ± 0.40	15.0 ± 0.49	15.8 ± 0.50	12.7 ± 0.39	0.39	0.34	0.03	< 0.01	<0.01	<0.001
IL-8	7.3 ± 1.02	8.1 ± 0.69	6.3 ± 1.26	7.8 ± 0.52	6.2 ± 0.48	0.54	0.27	0.94	0.13	0.93	0.04
IL-10	7.6 ± 0.24	7.5 ± 0.27	7.4 ± 0.22	7.4 ± 0.25	5.6 ± 0.30	0.81	0.74	0.78	< 0.001	<0.001	<0.001
TNF-α	11.0 ± 0.17	11.2 ± 0.21	11.7 ± 0.22	12.0 ± 0.26	11.5 ± 0.23	0.32	0.03	< 0.01	0.18	0.46	0.17
TGF-β	15.5 ± 0.17	15.4 ± 0.22	15.9 ± 0.29	16.0 ± 0.19	14.7 ± 0.24	0.79	0.07	0.04	< 0.01	<0.01	<0.01
***Enzymes***											
5-LO	17.9 ± 0.26	17.3 ± 0.26	14.1 ± 1.03	16.6 ± 0.93	15.3 ± 0.31	0.28	< 0.001	0.43	< 0.001	0.31	0.14
iNOS	6.2 ± 0.45	7.1 ± 0.54	6.5 ± 0.57	6.8 ± 0.49	4.2 ± 0.30	0.24	0.83	0.81	< 0.001	<0.01	<0.001

^1^ LAPH, dogs with leptospirosis and associated pulmonary hemorrhage and acute kidney injury; nLAPH, dogs with leptospirosis, but without associated pulmonary hemorrhage and with acute kidney injury; AKInL, dogs with acute kidney injury of other origin than leptosprosis (leptospirosis-negative dogs); CKD, dogs with chronic kidney disease; HC, healthy control dogs.

^2^ IL-1α, interleukin-1alpha; IL-1β, interleukin-1beta; IL-8, interleukin-8; IL-10, interleukin-10; TNF-α, tumor necrosis factor alpha; TGF-β, transforming growth factor beta; 5-LO, 5-lipoxygenase; iNOS, inducible nitric oxide synthase.

**Table 4:** Mean ± SEM of mRNA abundance (CT, log_2_) of genes encoding cytokines and enzymes in blood of diseased dogs one day before or at the day of death compared to diseased dogs that survived corresponding in time.

	Disease group^1^						ANOVA (*P*-value)	
	LAPH		AKInL		nLAPH	LEPTO		
	died	survived	died	survived	survived	survived	LAPH died	LAPH died
Variable^2^	(n = 9)	(n = 13)	(n = 4)	(n = 10)	(n = 11)	(n = 24)	vs. LAPH surv	vs. AKInL died
***Cytokines***								
IL-1α	13.1±0.40	12.3±0.38	13.1±0.52	12.7±0.46	12.4±0.39	12.3±0.27	0.19	0.92
IL-1β	16.1±0.53	14.3±0.41	16.5±0.53	15.1±0.48	14.7±0.46	14.4±0.30	0.01	0.61
IL-8	8.1±0.39	7.2±1.07	8.7±0.41	6.3±1.33	8.3±0.79	7.7±0.67	0.46	0.39
IL-10	7.5±0.37	7.6±0.27	6.8±0.87	7.4±0.21	7.5±0.32	7.6±0.20	0.72	0.42
TNF-α	10.0±0.26	11.1±0.19	10.7±0.71	11.7±0.20	11.2±0.29	11.2±0.16	< 0.01	0.30
TGF-β	14.9±0.35	15.6±0.18	15.3±0.51	16.0±0.31	15.4±0.22	15.5±0.14	0.09	0.59
***Enzymes***								
5-LO	17.1±0.46	16.9±0.31	18.0±0.79	14.2±1.04	17.1±0.30	17.0±0.21	0.63	0.33
iNOS	8.2±0.47	6.3±0.42	7.4±1.05	6.7±0.62	7.3±0.50	6.8±0.33	< 0.01	0.45

^1^ LAPH, dogs with leptospirosis and associated pulmonary hemorrhage and acute kidney injury; AKInL, dogs with acute kidney injury of other origin than leptospirosis (leptospirosis-negative dogs)

^2^ IL-1α, interleukin-1alpha; IL-1β, interleukin-1beta; IL-8, interleukin-8; IL-10, interleukin-10; TNF-α, tumor necrosis factor alpha; TGF-β, transforming growth factor beta; 5-LO, 5-lipoxygenase; iNOS, inducible nitric oxide synthase.

**Fig 1:**
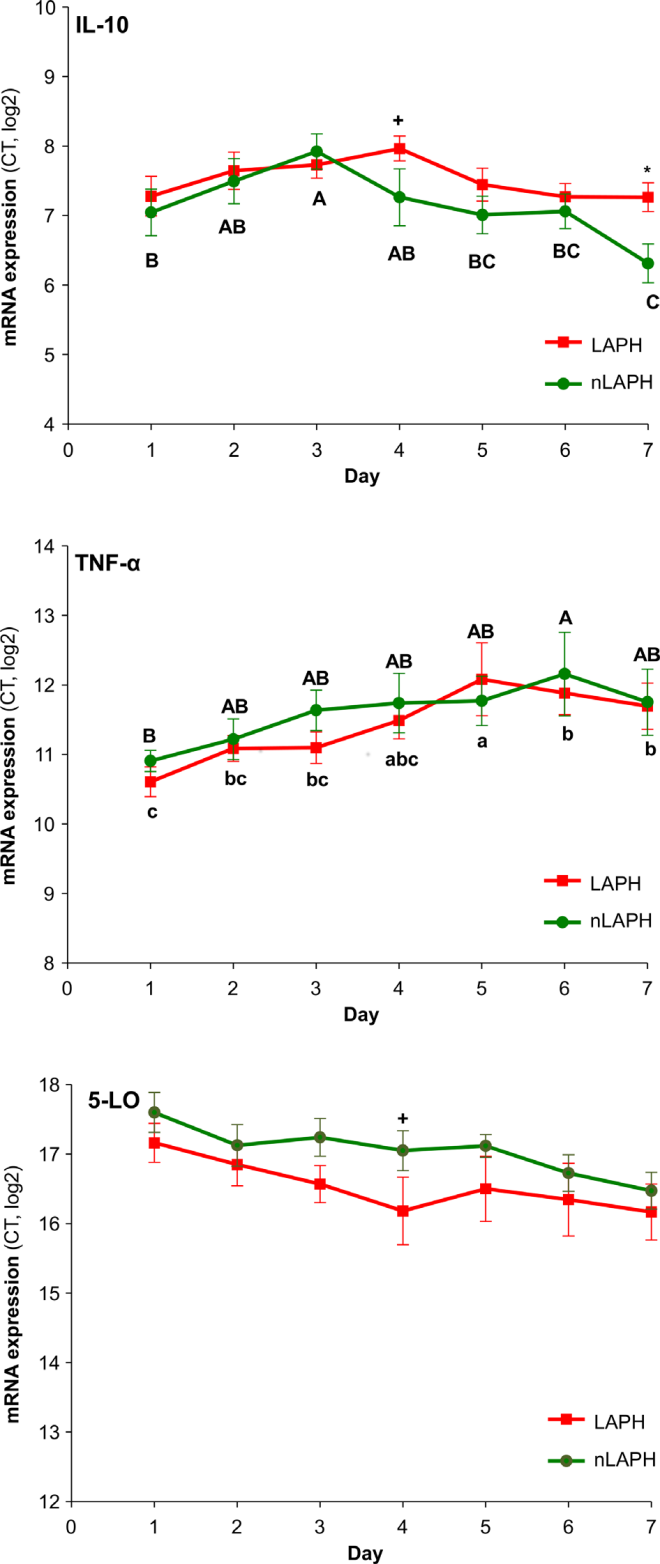
Time course of IL-10, TNF-α and 5-LO expression (mean ± SEM) in 24 survivor dogs with acute leptospirosis, including 13 dogs with (group LAPH) and 11 dogs without LAPH (group nLAPH). Differences between the disease groups LAPH and nLAPH are indicated as **+** (*P* < 0.1) and * (*P* < 0.05). Different letters indicate significant differences between the days (a,b,c for the LAPH group; A,B,C for the nLAPH group). Statistical significance. IL-10: disease effect *P* = 0.02, time effect *P*<0.01, and interaction *P* = 0.42; TNF-α: disease effect *P* = 0.35, time effect *P*<0.01, and interaction *P* = 0.94; 5-LO: disease effect *P* = 0.01, time effect *P* = 0.09, and interaction *P* = 0.98.

Furthermore, the mean ± SEM of RNA abundance (CT, log_2_) was calculated for each of the first 7 days after admission to the clinic for dogs with LAPH or nLAPH (Fig 1). The data were subsequently analyzed with a MIXED procedure of SAS (2001), including disease group and day after admission (day 1 to 7) as fixed effects. The day after admission was treated as repeated factor within subjects (animals). Multiple comparisons between the means at each sampling time-point were performed by the Bonferroni’s t-test, and differences between means were considered significant if *P* < 0.05.

## Results

### Characteristics of Included Animals

A total of 66 dogs were included in the study and divided into the four main disease groups according to the clinical diagnosis as described above. Leptospirosis was diagnosed in 34 animals (group LEPTO). The 22 dogs diagnosed with LAPH (subgroup LAPH) had a median age (interquartile range, IQR) of 5.3 (0.3–12.4) years; the group included 16 (73%) males and 6 (27%) females, and 13 (59%) dogs survived to discharge. The subgroup nLAPH included 12 dogs with a median age (IQR) of 4.6 (0.8–12.2) years, 8 (67%) were males and 4 (33%) females, and 11 (92%) dogs survived.

Acute kidney injury not due to leptospirosis was diagnosed in 14 dogs (group AKInL). The median age (IQR) was 3.7 (0.2–12.2) years; the group included 5 (36%) males and 9 (64%) females, and 10 (71%) dogs survived. The CKD group consisted of 8 dogs with a median age (IQR) of 7.2 (0.8–10.6) years, 5 (63%) males and 3 (38%) females. The HC group included 10 healthy dogs with a median age (IQR) of 3.1 (0.9–11.1) years, 4 (40%) males and 6 (60%) females.

Main clinical and laboratory characteristics of the dogs included in the study are summarized in Table 2.

### Expression Patterns of Cytokine and Enzyme mRNA for the Disease Groups during the First Three Days

The comparison of the areas under the curves of the mRNA expression of target cytokines and enzymes during the first three days after admission indicated that, compared to healthy dogs (HC), survivors of all disease groups showed elevated expression for IL-1α, IL-1β, IL-10, TGF-β and iNOS (*P* < 0.01). Interleukin-8 was higher expressed only in CKD dogs (*P* = 0.04) and 5-LO expression was elevated only in the LEPTO group (*P* < 0.001, Table 3). During the same initial time period, LEPTO dogs (LAPH and nLAPH) showed higher 5-LO (*P* < 0.001), lower TNF-α (*P* < 0.05), and a trend for lower TGF-β (*P* = 0.07) expression compared to AKInL. Decreased TNF-α expression (*P* < 0.01) was, however, also seen when compared to CKD dogs. Lower IL-1β and TGF-β expression (*P* < 0.05), and a trend to lower IL-1α expression (*P* < 0.1) were also noticed in LEPTO compared to CKD dogs. No significant difference was seen between LAPH and nLAPH for the measured cytokines and enzymes.

### Gene Expression Profiles of IL-10, TNF-α and 5-LO across Seven Days

The time course of the expression of the eight target genes was compared between survivors of LAPH and nLAPH over 7 days after admission to the clinic (Fig 1). The mRNA expression of IL-10 was affected by disease type (*P* < 0.05). It tended to be higher (*P* < 0.1) on day 4, and was higher (*P* < 0.05) on day 7 for LAPH dogs. In addition, the mRNA expression of IL-10 was affected by the day after admission to the clinic (*P* < 0.05), and was highest on day 3 compared to day 1, 5, 6, and 7 in nLAPH dogs.

No difference could be observed for TNF-α expression between the two disease groups across the seven days. However, the mRNA abundance of TNF-α changed over time in both LAPH and nLAPH dogs (*P* < 0.01). In LAPH dogs, the highest abundance was on day 5 compared to day 6 and 7 (*P* < 0.05). In nLAPH dogs, highest mRNA abundance of TNF-α was observed on day 6 compared to day 1 (*P* < 0.05).

An effect of disease type (*P* < 0.05) was observed for mRNA abundance of 5-LO which was observed on day 4 with a lower expression in LAPH. Furthermore a tendency for a time effect was observed (P < 0.1). No difference between the two disease groups was observed for the other five target genes.

### mRNA Expression of IL-1β, TNF-α and iNOS Is Associated with Mortality in LAPH

Gene expression in dogs with LAPH not surviving their disease was lower immediately before death for TNF-α (*P* < 0.01) and higher for IL-1β (*P* = 0.01) and iNOS (*P* < 0.01), compared to survivors of the same group at a corresponding time (Table 4). A trend to lower TGF-β (*P* = 0.09) was also observed in non-survivors. On the other hand no difference in mRNA expression was noted between non-survivors of the LAPH group and non-survivors of the AKInL group, possibly suggesting that this pattern is associated more with mortality from AKI in general than with mortality from LAPH in particular. With a very low number of dogs not surviving AKInL, this study was not powered to analyze this aspect further.

## Discussion

Investigation of the immune mechanisms involved in infection-related organ injury can be performed either by measuring the serum concentration of putative cytokines or by assessing their expression in the affected organs or in circulating leukocytes. By quantifying the protein itself, the assay of a cytokine concentration may be more predictive of its effect, although it does neither evaluate its biological activity at the tissue level, nor the effect of potential inhibitors [27]. The limited availability of validated canine assays restricts the spectrum of cytokines that can be assessed. Quantitative evaluation of cytokine expression by real-time PCR enables the analysis of samples with very low mRNA expression and it can be a valuable and sensitive tool to establish disease-related cytokine profiles [28]. The expression of mRNA does not necessarily reflect the actual cytokine concentration but, being the result of the upstream path of activation leading to gene expression, it offers valuable insights into the pathogenesis of inflammatory conditions [29].

A limited number of studies have addressed cytokine and enzyme expression in animal or human leptospirosis. A pattern of increased expression of IL-1α, IL-10, TNF-α, and COX-2 in blood was associated with lethal outcome in a hamster model [18]. The same model demonstrated renal expression of IP-10, IL-10, TNF-α, and TGF-β and a weak correlation with kidney damage [30]. Leptospirosis-susceptible hamsters showed strong expression of IL-1β, IL-6, TNF-α, and COX-2 in infected organs, whereas leptospirosis-resistant mice showed accelerated expression of the anti-inflammatory IL-10 [31].

Various human studies shed light on the immune response to leptospirosis in this species. High levels of IL-8 were reported in leptospiral hepatitis [32], TNF-α was related to kidney, liver, and lung involvement as well as to poor prognosis [33], and a high IL-10:TNF-α ratio was associated with mild disease [34] or with fatal outcome [17]. The levels of IL-1β, IL-6, IL-8, IL-10, and TNF-α were higher in non-survivors than in survivors, and IL-6 and IL-8 were associated with disease severity and mortality [35]. A more recent study showed higher IL-1β, IL-2. IL-4, IL-6, IL-8, IL-10, IL-17A, and TNF-α levels in severe leptospirosis compared to mild disease, and an association between high IL-6, IL-8, IL-10 and fatal outcome [36].

In the present study we described for the first time the mRNA expression profile of eight cytokines and enzymes involved in the immune response to naturally-occurring canine leptospirosis. We identified patterns associated with renal disease in general, with leptospirosis, with LAPH, and with fatal outcome of LAPH, possibly improving our prognostic ability and our understanding of the pathophysiological mechanisms of this severe infection for dogs and humans.

Dogs from all groups of renal diseases showed elevated expression of IL-1α, IL-1β, IL-10, TGF-β and iNOS, a pattern that may therefore be considered the signature for canine renal disease. Interleukin-1 is a pro-inflammatory acute phase cytokine with pyrogenic activity, acting synergistically with TNF-α and IL-6 [37,38,39,40]. IL-1α remains mostly cytosolic with autocrine activity or it is bound to the cell surface and released with cell necrosis, while IL-1β is circulating in the bloodstream [39]. In the present study, IL-1α and IL-1β were higher expressed in surviving dogs with all types of renal diseases compared to healthy dogs. Their highest expression in dogs with CKD possibly indicates a link with development of CKD.

Interleukin-10 and TGF-β are anti-inflammatory cytokines. IL-10 has pleiotropic effects, down-regulating innate and adaptive immune mechanisms, possibly preventing exaggerated immune responses [41,42]. The high expression of IL-10 in all renal disease groups compared to healthy dogs is consistent with observations in humans in which uremia was reported to influence expression of both pro- and anti-inflammatory cytokines, including IL-10 [43]. TGF-β controls iNOS expression, inhibits the activation of phagocytes, and promotes fibrosis in many diseases and organs, including the kidney [44,45]. Our observation of increased expression of TGF-β in AKI and highest expression in CKD could be associated with the reported risk of fibrosis and progression to chronic-on-acute kidney disease [46].

Inducible nitric oxide synthase (iNOS) generates nitric oxide (NO) from L-arginine, producing an effector molecule of the innate immune system to inhibit pathogen replication [47,48]. Nitric oxide is also closely involved in renal fibrosis and in the regulation of renal function, including glomerular filtration, tubular reabsorption, and renin secretion [49,50]. Decreased NO concentration has been associated with increased cardiovascular risk in human CKD, possibly due to the presence of uremia-associated iNOS inhibitors [51]. In a remnant kidney model of CKD, declining iNOS activity has been shown to parallel the progressive loss of renal function [52]. High serum NO concentrations have been shown in humans with severe leptospirosis [53] and a mouse and hamster model of leptospirosis indicated higher mortality, increased leptospiral burden and severe renal lesions after iNOS inhibition [54]. Increased iNOS expression seems therefore to be a feature of leptospirosis where it could play a protective role. Increased NO production has also been reported to induce local and systemic oxidative damage in other renal diseases [55]. In the present study, increased iNOS gene expression in blood leukocytes in all dogs with renal diseases suggests a role not restricted to leptospirosis alone.

Dogs with leptospirosis were characterized by a high 5-LO and low TNF-α expression compared to other dogs with AKI, although the decreased TNF-α expression was also seen in dogs with CKD. 5-LO is the key enzyme converting arachidonic acid into leukotrienes and 5-hydroxyeicosatetraenoic acid [56,57]. It is expressed primarily in inflammatory cells, interacts with receptors on airway smooth muscle and vascular endothelium, and is therefore key in the control of vascular, bronchial, hepatic, and systemic inflammation [56,58,59,60]. The high expression of 5-LO in canine leptospirosis could thus represent one of the links between the target organs of this multisystemic infection. To the authors’ knowledge, no research has been done previously on the role of 5-LO in leptospirosis.

Interestingly, dogs with LAPH were not characterized by a specific cytokine signature and they only displayed minimal differences with increased IL-10 and decreased 5-LO expression at some time points despite marked differences in the clinical manifestations. This could be explained by the limited number of cytokines and enzymes evaluated in the present study and other molecules of interest could include cytokine receptors, chemokines, or complement factors. In humans for example, soluble ST2, a member of the IL-1 receptor family, was associated with bleeding in severe leptospirosis [61]. The possibility that all dogs with leptospirosis were affected with pulmonary hemorrhages of different grades, from subclinical to severe, and that the radiological distinction used in this study would therefore be arbitrary, should also be considered.

In the present study, low TNF-α, high IL-1β, and high iNOS expression seemed to be associated with fatal outcome during the course of LAPH. Surprisingly, however, mortality was not associated with increased IL-10 expression, unlike results from studies in hamsters and humans [18,36]. Mortality in clinical veterinary studies being influenced by the owners’ perception of the disease and their decision to euthanize severely affected animals, it is not only a reflection of very severe clinical manifestation. However, fatal course of LAPH is typically rapid and most dogs euthanized in this study would not have survived even with intensive therapy.

Aside from the relatively small number of cases included in some of the groups, one of the major limitations of this study is related to the naturally-occurring model of infection. In contrast to an experimental inoculation [18], the day of infection could not be determined exactly, making it difficult to compare time courses of cytokine and enzyme expression between dogs. Pre-referral treatment differed between dogs and it is likely that the infection varied in terms of infecting serovars and strains, route of infection, and size of inoculum These disadvantages of a clinical study are however offset by the fact that it reflects more precisely the reality of the animals diagnosed and treated for leptospirosis.

In summary, this study shows that dogs with naturally-occurring leptospirosis display a cytokine and enzyme pattern characterized by higher 5-LO and lower TNF-α expression than dogs with AKI from other origin. Dogs with LAPH were not characterized by a particular pattern, but their outcome was worse with low TNF-α, high IL-1β, and high iNOS expression. Although sometimes different from patterns previously described in other animal species and humans, the cytokine and enzyme signatures observed in these dogs with severe leptospirosis indicate a complex pro- and anti-inflammatory response to the leptospires. In addition to a role as biomarkers of disease activity, the identified cytokines and enzymes likely play an active role in the development or the modulation of the various clinical manifestations of the disease, and this remains to be evaluated with pharmacological interventions or blood purification techniques targeting the identified immune pathways.

## Supporting Information

S1 File Manuscript “Evidence of uremic inflammation in dogs with renal disease” by Nentwig et al. This manuscript evaluated the evidence of uremic inflammation the same dogs as the present manuscript. It has been accepted for publication in the American Journal of Veterinary Research (2016).
(PDF)

S1 Table List of the individual dogs, their grouping and their cytokine expression for the first 3 days of hospitalization. (PDF)
