# Supplementary material for: Expression Profile of Cytokines and Enzymes mRNA in Blood Leukocytes of Dogs with Leptospirosis and Its Associated Pulmonary Hemorrhage Syndrome
Source: PLoS One. 2016 Jan 29;11(1):e0148029. doi: 10.1371/journal.pone.0148029 (PMC4732604; doi:10.1371/journal.pone.0148029)
Supplement: S1 File — This manuscript evaluated the evidence of uremic inflammation the same dogs as the present manuscript. It has been accepted for publication in the American Journal of Veterinary Research (2016). (PDF) [file pone.0148029.s001.pdf]

Evidence of uremic inflammation in dogs with renal disease

Authors

Alice Nentwig, Dr med vet; Ariane Schweighauser, Dr med vet; Carla Maissen-Villiger, Dr med vet; Rupert M. Bruckmaier, Dr med vet; Andreas Zurbriggen, Dr med vet; Anette H. van Dorland, Dr sc ETH; Thierry Francey, Dr med vet

From the Department of Clinical Veterinary Medicine, Division of Small Animal Internal Medicine (Nentwig, Schweighauser, Maissen-Villiger, Francey), the Division of Veterinary Physiology, Vetsuisse Faculty University of Berne, Switzerland (Bruckmaier), the Division of Neurological Sciences (Zurbriggen), Vetsuisse Faculty University of Berne, Switzerland; and the University of Applied Sciences, Zollikofen, Switzerland (van Dorland). Dr. Maissen-Villiger's present address is Kleintierpraxis AMC, Murten, Switzerland.

Address correspondence to Dr. Nentwig (alice.nentwig@vetsuisse.unibe.ch)

Abstract

Objective – To assess the inflammatory status in dogs with uremia by measuring the expression of inflammatory cytokines and enzymes in venous whole blood.

Animals – 46 dogs with acute kidney injury (AKI), 8 dogs with chronic kidney disease (CKD) and 10 healthy dogs.

Procedures – Dogs with AKI and CKD were prospectively enrolled during the year 2010 if they met the inclusion criteria. Demographic and laboratory characteristics were evaluated for every dog and the expression of inflammatory cytokines (IL-1α. IL-1β, IL-8, TNF-α, IL-10, TGF-β) and enzymes (iNOS, LO5) measured at initial presentation using venous whole blood.

Results – Azotemic dogs had marked increases in the expression of the cytokines IL-1α, IL-1β, TGF-β and the enzyme LO5 when compared to healthy dogs. Additionally 36.4% of AKI dogs (but none of the CKD dogs) had higher IL-8 mRNA levels and 37.5% of CKD dogs (but only 4.4% of AKI dogs) had higher TNF-α levels when compared to healthy dogs. No significant difference could be detected between the inflammatory markers and laboratory characteristics, degree of azotemia or etiology of the kidney injury.

Conclusions and Clinical Relevance – Expression of the cytokines IL-1α, IL-1β, TGF-β and the enzyme LO5 were clearly increased in azotemic dogs, suggesting that these markers are part of an inflammatory response seen in animals with acute or chronic kidney injury.

Abbreviations

AKI Acute kidney injury

CKD Chronic kidney disease

IL Interleukin

iNOS inducible nitric oxide synthase

IRIS International renal interest society

LO5 5-lipoxygenase

TGF Transforming growth factor

TNF Tumor necrosis factor

RT-PCR Real-time polymerase chain reaction

Introduction

Uremia is typically defined as the complex signs and clinical symptoms that result as a consequence from loss of kidney function. Uremia is not only caused by the accumulation of organic waste products, but also by impaired metabolic and endocrine renal functions. The most common cause of uremia is CKD, but also other conditions such as AKI, lower urinary tract obstruction and rupture of the urinary tract may occasionally cause it[1]. In human medicine it has been clearly shown in patients with CKD that persistent chronic inflammation is a major contributing factor for increased morbidity and mortality[2]. Factors contributing to this inflammatory process are a retention of mediators such as circulating cytokines and pro-oxidants with declining kidney function, but also increased tissue production of inflammatory mediators caused by uremia itself, oxidative stress and other factors such as overhydration[2,3]. In human medicine AKI occurs in 1% of hospital admissions and up to 7% of hospitalized patients develop AKI[4]. In veterinary medicine, the incidence of AKI in hospital admissions is

unknown, but studies investigating hospital acquired AKI described a prevalence between 12-14.6%[5,6]. It has been shown that the extent of the increase in azotemia can be used as a predictor of mortality in dogs and cats[7]. Despite the availability of both intermittent hemodialysis and continuous renal replacement therapy, mortality still remains high both in humans and animals. In a recent study of cats and dogs with AKI treated with IHD it was demonstrated that although there was a high mortality rate prior to discharge, dogs had an overall survival rate to discharge of 53%, similar to numbers reported in human patients[8].

It is now believed, that inflammation plays also a major role in the pathophysiology of AKI, not only in the initiation but also in the extension phase. It is thought that the initial insult on the kidneys results in morphological and functional changes in vascular endothelial cells and the tubular epithelium. Subsequently renal endothelial cells and proximal tubular epithelial cells produce cytokines and chemokines that lead to infiltration of inflammatory cells such as neutrophils, lymphocytes, macrophages and natural killer cells into the kidney. Inflammatory cells then further produce pro- and anti-inflammatory cytokines that may contribute to the already existing inflammation[4].

The term pro-inflammatory cytokines includes many cytokines characterized by being inducible and belonging to different families, including IL-1, IL-2, IL-6, IL-8, members of the TNF family, IFN-γ and others. Anti-inflammatory cytokines, such as IL-10 or TGF-β typically control or decrease inflammatory processes[9].

Several studies about AKI in experimental settings in mice or rats, but also clinical studies in human patients with AKI demonstrated increased levels of pro- and anti-inflammatory cytokines and mediators. In experimental studies in mice with ischemic AKI it could be demonstrated that IL-2, IL-6, IL-10, IFN-γ and TGF-β are, amongst others, increased in the kidney tissue[10,11]. Similar results have been obtained in another study with elevated levels of IL-1α, IL-1β, IL-6 and IL-18 in the kidney tissue after cisplatin-induced AKI in mice[12]. Fewer studies evaluated inflammatory mediators in the human population after AKI. Simmons et

al.[13] detected that patients with AKI had significantly higher plasma levels of IL-1β, IL-6, IL-8, TNF-α, CRP and IL-10 compared to healthy people and patients with CKD. Additionally IL-6 and IL-8, but also the anti-inflammatory IL-10 were significantly higher in nonsurvivors. In other studies it was shown that IL-2, IL-6 and TNF-α were markedly higher in patients with AKI and were also significantly related with mortality rates[14,15].

LO5 is primarily expressed in leukocytes and is the key enzyme in leukotriene biosynthesis, which are considered potent mediators of inflammation[16]. The LOX enzymes have also been implicated in renal vasoconstriction and inflammation in AKI and lipoxygenase interaction products (lipoxins) have shown to be beneficial in AKI[17,18]. One of the key enzymes generating nitric oxide from l-arginine is iNOS. iNOS-derived nitric oxide plays an important role in inflammatory conditions. In rats an increase in iNOS simultaneously with the major damage to the kidney was evidenced after induced ischemic AKI[19].

We hypothesized first, that based on human medicine and experimental studies uremia is an inflammatory condition in dogs. Second, that the degree of inflammation correlates with the severity of azotemia and third, that the degree of inflammation is more pronounced in dogs with acute kidney injury compared to dogs with chronic kidney disease.

Materials and Methods

Between the study period from January 1, 2010 to December 31, 2010 all dogs presenting with azotemia due to either AKI or stable CKD to the small animal clinic of the Vetsuisse faculty in Bern, Switzerland were considered for this prospective study. All dogs enrolled in this study were also part of another study that focused on the dogs with leptospirosis-associated pulmonary hemorrhage syndrome. [a] AKI was defined as the occurrence of acute onset of renal azotemia and compatible historical, clinical, clinicopathological and

radiological findings. CKD was defined as presence of stable azotemia for at least 3 months duration, inability to concentrate urine and compatible findings in abdominal ultrasonography. Exclusion criteria included known or suspected acute-on-chronic kidney disease, unstable CKD, glomerulonephritis, pre- and postrenal causes of azotemia. 46 dogs met the entry criteria for AKI, and 8 for CKD. In this population, AKI was mainly due to leptospirosis (69.6%), but also grape ingestion (6.5%), other intoxications (4.3%) and AKI of unknown origin (19.6%). Dogs with AKI were classified into five different grades as proposed by Cowgill[20]. AKI grade I consisted of dogs with non azotemic AKI (serum creatinine < 140 µmol/l), grade II consisted of dogs with mild AKI (serum creatinine 141-220 µmol/l) and grade III-V consisted of dogs with moderate to severe AKI (grade III: serum creatinine 221-445 µmol/l; grade IV: serum creatinine 446-890 µmol/l; grade V: serum creatinine > 890 µmol/l). Dogs with stable CKD were classified into 4 different stages as defined by the IRIS[21]. IRIS stage I consisted of dogs with non-azotemic CKD (serum creatinine < 125 µmol/l), IRIS stage II consisted of dogs with mild renal azotemia (serum creatinine 125-179 µmol/l), IRIS stage III consisted of dogs with moderate renal azotemia (serum creatinine 180-439 µmol/l) and IRIS stage IV of dogs with severe renal azotemia (serum creatinine > 440 µmol/l).

Ten clinically healthy control dogs from staff or volunteering owners were also evaluated. Their health evaluation included physical examination, complete blood count, chemistry, and urinalysis.

Demographic and laboratory characteristics (complete blood count, chemistry, urinalysis) were evaluated for every dog. For the purpose of this study, the expression of the anti-inflammatory cytokines IL-10 and TGF-β, as well as the pro-inflammatory cytokines IL-1α, IL-1β, IL-8 and TNF-α were measured at initial presentation using venous whole blood. Additionally the enzymes LO5 and iNOS were also evaluated. Analysis of each cytokine and

enzyme was done measuring mRNA expression by RT-PCR as described in detail previously.[a] Shortly, mRNA was extracted from whole blood using a commercially available kit[b], according to the manufacturer's protocol. Before RT-PCR, RNA was converted to cDNA using a synthesis kit[c]. Specific primers were used as previously described.[a] . The RT-PCR assay was developed and evaluated on a real-time DNA detection system[d]. mRNA levels were calculated relative to the housekeeping gene ubiquitin, which was used as a control. This study was approved by the animal experimental ethics committee of the Small animal teaching hospital of the Vetsuisse faculty University of Bern.

The statistical analysis was performed with a commercial software.[e] Since most data were not normally distributed, non-parametric statistics were used. Continuous data were compared with the Mann-Whitney rank-sum test. Regression analysis was performed using the Spearman's rank correlation. The proportion of dogs with higher inflammatory biomarker level was established comparing individual values of AKI/CKD dogs to the range detected in healthy dogs. $P \leq 0.05$ was considered significant.

Results

The dogs in the AKI group ranged in age from 2 months to 12.3 years (median, 4.8 years), the dogs in the CKD group ranged from 9 months to 10.6 years (median, 6.5 years) and the dogs in the healthy group ranged in age from 1 to 11.1 years (median, 3.9 years). In the AKI group, one dog was classified as grade I, 10 dogs as grade IV and 16 dogs as grade V. In the CKD group, one dog was classified as IRIS stage 3 and the remaining seven dogs as IRIS stage 4. Baseline clinicopathological characteristics for all dogs are presented in table 1. Packed cell volume was markedly higher in AKI compared to CKD, healthy dogs compared

to AKI and healthy dogs compared to CKD. The platelets were markedly lower in AKI compared to healthy dogs. White blood cell counts were elevated in AKI compared to CKD and healthy dogs. Urea and creatinine were also markedly elevated in AKI compared to healthy dogs and in CKD compared to healthy dogs.

Azotemic dogs (AKI and CKD) had marked increases in blood mRNA levels of the cytokines IL-1α ($P < 0.00004$), IL-1β ($P < 0.00097$), TGF-β ($P < 0.0049$) and the enzyme LO5 ($P < 0.004$) compared to healthy dogs. The increase of TGF-β compared to healthy dogs could only be seen in dogs with AKI, but not in dogs with CKD. No significant difference could be seen in the cytokine and enzyme gene expression between AKI and CKD dogs (figure 1). However dogs with AKI displayed more variability in their expression of most cytokines and enzymes, in particular for IL-1α, IL-8, LO5, and iNOS. No obvious correlation could be detected in the regression analysis in azotemic dogs in general (CKD and AKI dogs together) and in the group of CKD and AKI dogs alone between IL-1α and serum creatinine concentration (figure 2). Additionally in AKI dogs, no correlation could be seen if the different etiologies were looked at separately and infectious causes such as leptospirosis were compared to non-infectious causes of AKI. For a better assessment of a possible correlation between the inflammatory markers and the degree of azotemia, CKD and AKI dogs were additionally divided into quartiles (according to their creatinine and urea value) and also grouped according to their stage or grade of CKD or AKI, respectively. No correlation between the degree of azotemia and the expression of the inflammatory markers could be detected with any of these classifications as can be seen in figure 3 for IL-1α.

Inflammatory biomarkers were elevated in 4.4 – 76.1% of dogs in the AKI group and 0-87.5% of dogs in the CKD group compared to healthy dogs (figure 4). 36.4% of the AKI dogs, but none of the CKD dogs had higher IL-8 mRNA levels compared to healthy dogs. This difference was statistically significant ($P = 0.047$). Also, significantly more CKD dogs

(37.5%) than AKI dogs (4.4%) had elevated levels of TNF-α (*P* = 0.02). Interestingly, none of the dogs showed lower inflammatory biomarker levels than healthy dogs.

Discussion

Based on these results, uremia seems to be an inflammatory condition with dysregulation of the measured cytokines and enzymes, both in dogs with stable CKD, but also in animals with AKI. The pro-inflammatory markers IL-1α, IL-1β, and LO5 as well as the anti-inflammatory marker TGF-β were all elevated in uremic dogs and TGF-β was also significantly increased in AKI compared to CKD. The IL-1 molecule comprises a major pro-inflammatory family of cytokines which acts mainly through the induction of pro-inflammatory cytokines and mediators. Studies have shown that the initial inflammatory response is induced by IL-1α derived from damaged cells. Subsequently resident macrophages respond to IL-1α and produce IL-1β which is then responsible for the propagation of the inflammatory response[22-24]. LO5 is implicated as an inflammatory enzyme in kidney injury, but it does not seem to be a commonly used biomarker of inflammation in patients with AKI[17]. Since LO5 was clearly higher in azotemic animals it might be useful as part of a biomarker panel, especially since lipoxins are proven to be beneficial as a treatment for ischemic AKI.

The kidney is a site of both TGF-β production and TGF-β action. It is well known that TGF-β plays a key role in chronic progressive renal disease not only mediating fibrogenesis but also apoptosis and epithelial-to-mesenchymal transdifferentiation[25]. Increased expression in our CKD population is therefore not surprising. In our animals with AKI, TGF-β was also significantly higher compared to CKD dogs. In an experimental rat model with induced ischemic AKI, TGF-β was elevated within 3 days and remained elevated for up to 7 days. The role of TGF-β in AKI is not completely clear, but TGF-β activity appears to influence cellular

proliferation after the initial insult, although clear evidence is lacking that TGF-β plays a substantial role in renal repair response. Contrary to that, TGF-β activity promotes renal fibrogenesis and renal blood vessel loss following AKI and therefore may predispose the kidney for the development of CKD[26].

It is unclear why other inflammatory cytokines such as TNF-α that have been implicated several times to be elevated in experimental models but also clinical studies with AKI were not different between azotemic and healthy animals in our study. Also, no correlation could be detected between individual laboratory parameters, especially degree of azotemia and cytokine gene expression. Additionally, with the exception of TGF-β no difference could be shown between dogs with AKI and CKD.

Potential explanations include the timing of cytokine determination in the overall course of disease. The time between onset of clinical signs and presentation to our hospital varied among dogs with AKI. It is therefore possible that differences in expression of early and late inflammatory markers may have been missed. It is also important to remember that inflammatory cytokine and enzyme biology is very complex in disease states and a lack of correlation between gene expression of cytokines and their bioactivity has been demonstrated[27]. Of course this hypothesis should be confirmed with the measurement of cytokine plasma levels. In some diseases, measurement of cytokines in other tissues than plasma might be useful. As an example, measurement of urinary cytokine IL-6 better reflects the disease state than circulating plasma IL-6[28] and also measurement of inflammatory cytokines in bronchial alveolar lavage fluid has better prognostic value than in plasma[29,30]. It also has to be taken into account that in the majority of experimental studies of AKI, cytokines were measured directly in the kidney tissue. This procedure is not without risk and therefore not as routinely performed in veterinary medicine as it is in human medicine. Maybe measuring cytokine expression in urine instead of plasma would have given us a clearer

picture about the inflammatory process in AKI, but since most of our dogs are oligo-anuric, this might not have been possible in most of the cases.

Other reasons might be, that the differences in plasma cytokine expression between the groups are too small to get detected by the methods used in this study or that variations are due to the heterogeneity of the disease groups. A similar observations has been made in another study for plasma levels of IL-1β[31]. Other limitations would be the sample size in our groups; however the power should be sufficient to detect major, clinically relevant differences. Another point is that the degree of inflammation is certainly multifactorial and caused by a combination of primary (etiology) and secondary (complications) parameters.

Since the inflammatory process is very complex it is possible that different biomarkers than the ones we used would better represent inflammation in AKI. IL-6 is an important pro-inflammatory cytokine in the kidney and its release is stimulated by TNF-α. In several studies, plasma IL-6 levels have been shown to be increased in patients with AKI and could also be used to predict mortality[13,15,31,32]. In addition, urinary IL-6 has been proposed as an early marker for acute renal allograft rejection[33]. Furthermore, IL-18 which is a pro-inflammatory cytokine induced in the proximal tubule after injury, seems to be a potential biomarker for AKI. Urinary IL-18 levels were not only increased in mice with ischemic AKI but also in human patients with acute tubular necrosis and delayed graft function compared with other renal diseases[34]. Several studies demonstrated, that IL-18 could not only be used as a good marker for early detection of AKI, but also to predict mortality risk after AKI[35].

In conclusion, a clear increase could be detected in the blood mRNA expression of the cytokines IL-1α, IL-1β, TGF-β and the enzyme LO5 in azotemic dogs, suggesting that these markers are part of an inflammatory response seen with acute or chronic kidney injury. Although no difference in expression could be observed between dogs with AKI or CKD, AKI dogs were more frequently expressing elevated IL-8 mRNA levels and CKD dogs elevated TNF-α levels, suggesting an association with the type of renal disease. Further

studies are needed to assess cytokine levels in other samples such as urine or renal parenchyma within the same animal groups. An extension of this panel of biomarkers and their sequential measurement over the time course of the disease might also help to better understand the inflammatory status of critically ill dogs with AKI.

a. Villiger-Maissen et al. Expression profile of cytokines and enzymes mRNA in blood leukocytes of dogs with leptospirosis-associated pulmonary hemorrhage syndrome. Submitted 2014

b. RNeasy® Protect Animal Blood Kit, Qiagen, Switzerland

c. SuperScript® VILO™ cDNA Synthesis Kit, Invitrogen, Switzerland

d. Rotor-Gene™ 6000, Corbett Research, Australia

e. NCSS software, Kaysville, Utah, USA

Figures and Tables

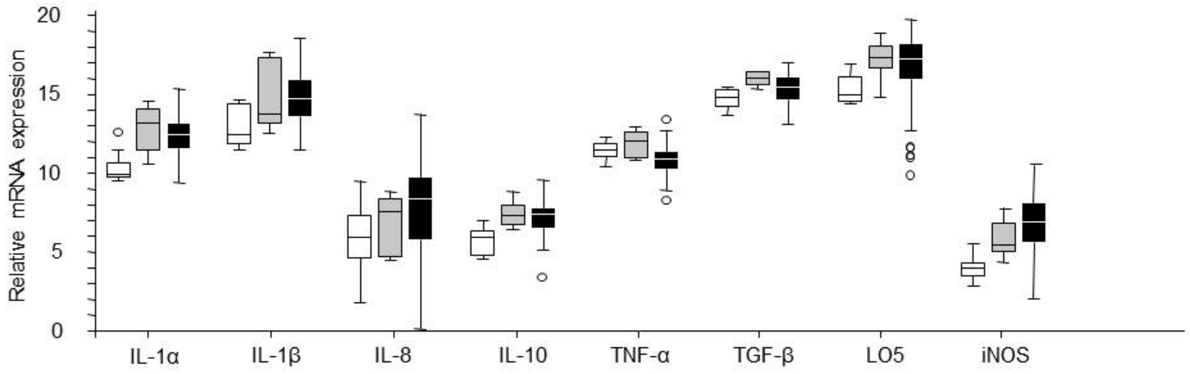

Figure 1. Relative mRNA expression of cytokines and enzymes in whole blood in dogs presenting with AKI (black), CKD (grey) and healthy dogs (clear).

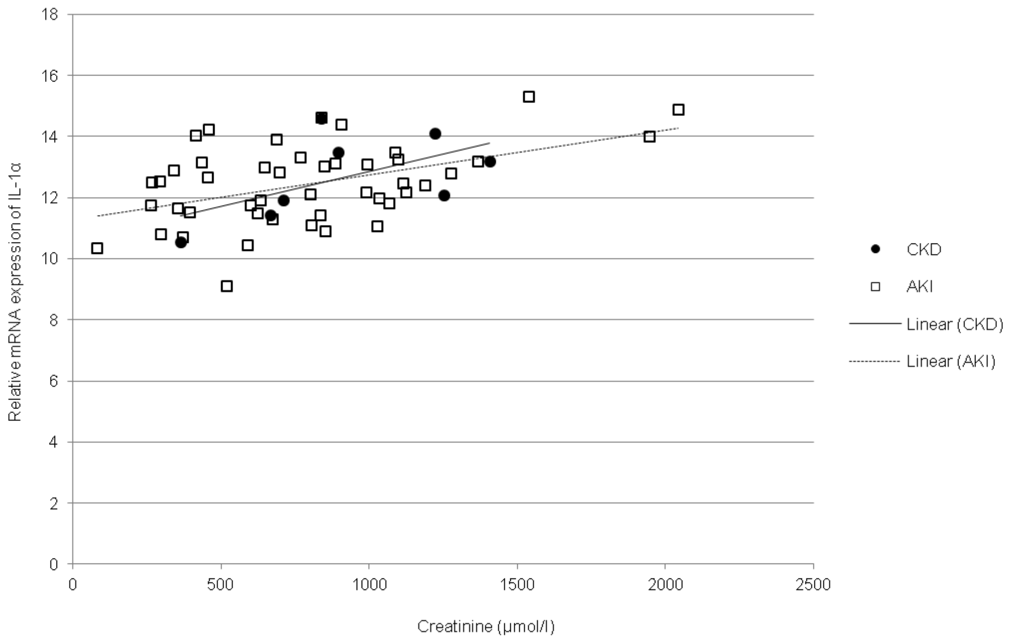

Figure 2. Relative mRNA expression of IL-1α in correlation to serum creatinine concentration (in µmol/l) in dogs with AKI (r^2 = 0.22, *P* = 0) and CKD (r^2 = 0.33, *P* = 0.14).

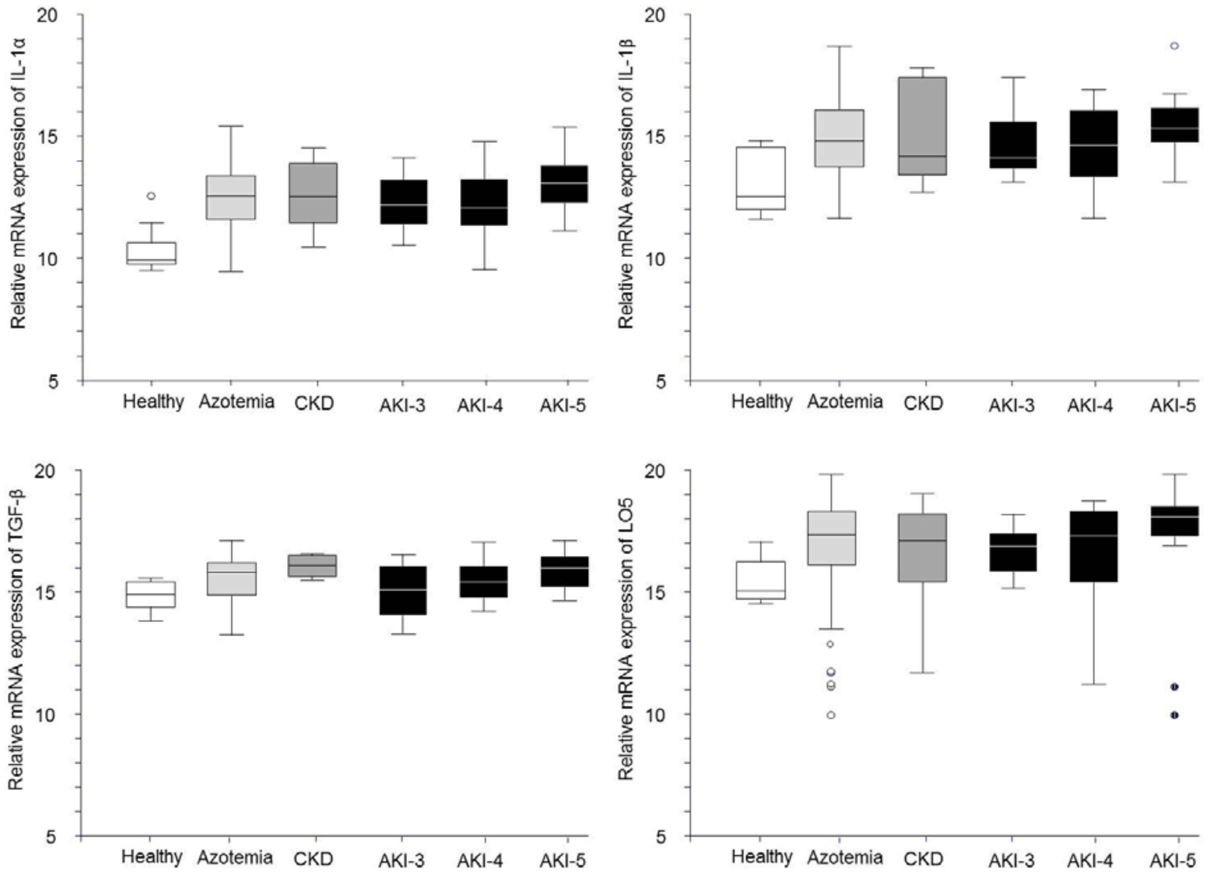

Figure 3. Relative mRNA expression of IL-1α, IL-1β, TGF-β and LO5 in healthy dogs (clear), dogs with azotemia (light grey), CKD (dark grey) and dogs with AKI according to their clinical grade (black). AKI-3, acute kidney injury grade 3; AKI-4, acute kidney injury grade 4; AKI-5, acute kidney injury grade 5.

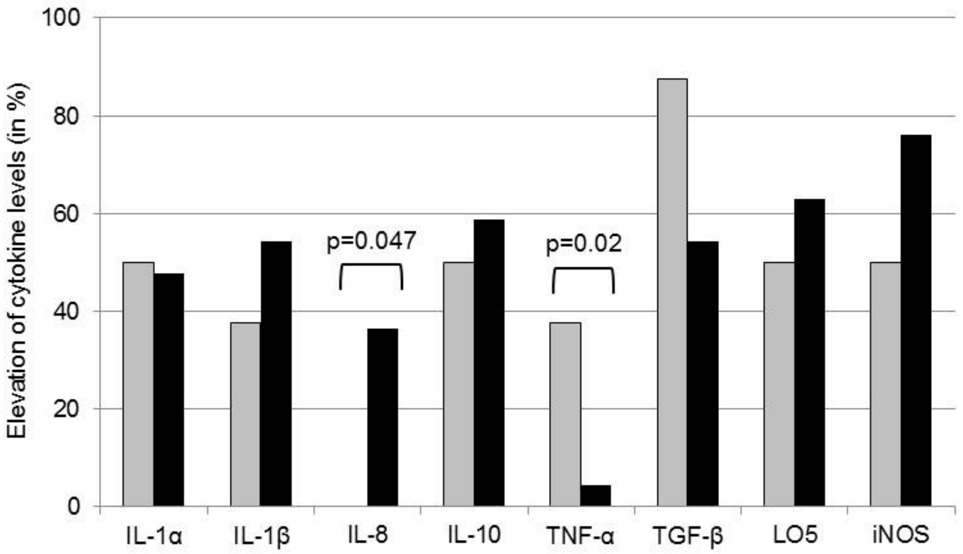

Figure 4. Proportions of dogs with AKI (black) and CKD (grey) that had higher cytokine levels compared to healthy dogs and statistical significance when comparing these two groups.

Table 1. Median and interquartile range of main laboratory characteristics of all dogs at presentation.

	AKI (n=46)	CKD (n=8)	Healthy (n=10)
PCV (%)	32.5 (28-40)[a]	23.5 (14.8-30.5)[a]	43 (41-46.5)[a]
Platelets (10^9/l)	166 (90.8-256.3)[b]	205.5 (101.8-261.8)	226 (202.3-278.5)[b]
WBC (10^9/l)	14.7 (11.7-19)[cd]	8.9 (7.1-13.8)[c]	11.2 (7.1-11.6)[d]
Creatinine (µmol/l)	785.5 (449.5-1044)[e]	866.5 (679-1246)[f]	80.5 (70.3-92.3)[ef]
Urea (mmol/l)	55.8 (39.7-73.1)[g]	77.4 (54.5-83.7)[h]	6.5 (4.6-7.2)[gh]

[a-h] For a given variable, values with same superscript letters differed significantly ($P < 0.05$).

PCV, packed cell volume; WBC, white blood cells.

References

1. Squires R. Uraemia. In: Elliott J, Grauer G, eds. BSAVA Manual of Canine and Feline Nephrology and Urology. 2nd ed. Gloucester: British Small Animal Veterinary Association, 2007;54-68.
2. Schindler R. Causes and therapy of microinflammation in renal failure. Nephrol Dial Transplant 2004;19:34-40.
3. Carrero JJ, Stenvinkel P. Inflammation in end-stage renal disease-what have we learned in 10 years? Semin Dial 2010;23:498-509.
4. Akcay A, Nguyen Q, Edelstein CL. Mediators of inflammation in acute kidney injury. Mediators Inflamm 2009:1-12.
5. Thoen M, Kerl M. Characterization of acute kidney injury in hospitalized dogs and evaluation of a veterinary acute kidney injury staging system. J Vet Emerg Crit Care 2011;21:648-657.
6. Kenney E, Rozanski E, Rush J, et al. Association between outcome and organ system dysfunction in dogs with sepsis: 114 cases (2003-2007). J Am Vet Med Assoc 2010;236:83-87.
7. Harison E, Langston C, Palma D, et al. Acute azotemia as a predictor of mortality in dogs and cats. J Vet Intern Med 2012;26:1093-1098.
8. Eatroff AE, Langston CE, Chalhoub S, et al. Long-term outcome of cats and dogs with acute kidney injury treated with intermittent hemodialysis: 135 cases (1997-2010). J Am Vet Med Assoc 2012;241:1471-1478.
9. Abbas A, Lichtman A, Pillai S. Cytokines. In, eds. Cellular and Molecular Immunology. 6th ed. Philadelphia, PA: Saunders Elsevier, 2007;267-301.
10. Goes N, Urmson J, Ramassar V, et al. Ischemic acute tubular necrosis induces an extensive local cytokine response. Evidence for induction of interferon-gamma,

transforming growth factor-beta 1, granulocyte-macrophage colony-stimulating factor, interleukin-2, and interleukin-10. Transplantation 1995;59:565-572.

11. Kielar ML, John R, Bennett M, et al. Maladaptive role of IL-6 in ischemic acute renal failure. J Am Soc Nephrol 2005;16:3315-3325.

12. Faubel S, Lewis EC, Reznikov L, et al. Cisplatin-induced acute renal failure is associated with an increase in the cytokines interleukin (IL)-1beta, IL-18, IL-6, and neutrophil infiltration in the kidney. J Pharmacol Exp Ther 2007;322:8-15.

13. Simmons EM, Himmelfarb J, Sezer MT, et al. Plasma cytokine levels predict mortality in patients with acute renal failure. Kidney Int 2004;65:1357-1365.

14. Ficek R, Kokot F, Chudek J, et al. Plasma concentrations of tumor necrosis factor alpha may predict the outcome of patients with acute renal failure. Kidney Blood Press Res 2006;29:203-209.

15. Kadiroglu AK, Sit D, Atay AE, et al. The evaluation of effects of demographic features, biochemical parameters, and cytokines on clinical outcomes in patients with acute renal failure. Ren Fail 2007;29:503-508.

16. Steinhilber D. 5-Lipoxygenase: a target for antiinflammatory drugs revisited. Curr Med Chem 1999;6:71-85.

17. Imig JD. Eicosanoids and renal vascular function in diseases. Clin Sci 2006;111:21-34.

18. Kieran NE, Maderna P, Godson C. Lipoxins: potential anti-inflammatory, proresolution, and antifibrotic mediators in renal disease. Kidney Int 2004;65:1145-1154.

19. Villanueva S, Cespedes C, Gonzalez AA, et al. Effect of ischemic acute renal damage on the expression of COX-2 and oxidative stress-related elements in rat kidney. Am J Physiol Renal Physiol 2007;292:1364-1371.

20. Cowgill L, Langston C. Acute Kidney Insufficiency. In: Bartges J, Polzin D, eds. Nephrology and Urology of Small Animals. 1st ed. Ames, IA: Blackwell Publishing Ltd, 2010;472-523.

21. International Renal Interest Society (IRIS) website. IRIS Staging of CKD. Available at: www.iris-kidney.com/guidelines/staging.shtml. Accessed Sept 15, 2014.

22. Rider P, Carmi Y, Guttman O, et al. IL-1alpha and IL-1beta recruit different myeloid cells and promote different stages of sterile inflammation. J Immunol 2011;187:4835-4843.

23. Dinarello CA. Interleukin-1 in the pathogenesis and treatment of inflammatory diseases. Blood 2011;117:3720-3732.

24. Ortega L, Fornoni A. Role of cytokines in the pathogenesis of acute and chronic kidney disease, glomerulonephritis, and end-stage kidney disease. Int J Interferon, Cytokine and Mediator Res 2010;2:49-62.

25. Böttinger EP, Bitzer M. TGF-beta signaling in renal disease. J Am Soc Nephrol 2002;13:2600-2610.

26. Spurgeon KR, Donohoe DL, Basile DP. Transforming growth factor-beta in acute renal failure: receptor expression, effects on proliferation, cellularity, and vascularization after recovery from injury. Am J Physiol Renal Physiol 2005;288:568-577.

27. Himmelfarb J, Le P, Klenzak J, et al. Impaired monocyte cytokine production in critically ill patients with acute renal failure. Kidney Int 2004;66:2354-2360.

28. Otto G, Braconier J, Andreasson A, et al. Interleukin-6 and disease severity in patients with bacteremic and nonbacteremic febrile urinary tract infection. J Infect Dis 1999;179:172-179.

29. Pittet JF, Mackersie RC, Martin TR, et al. Biological markers of acute lung injury: prognostic and pathogenetic significance. Am J Respir Crit Care Med 1997;155:1187-1205.

30. Pugin J, Ricou B, Steinberg KP, et al. Proinflammatory activity in bronchoalveolar lavage fluids from patients with ARDS, a prominent role for interleukin-1. Am J Respir Crit Care Med 1996;153:1850-1856.

31. Himmelfarb J, McMonagle E, Freedman S, et al. Oxidative stress is increased in critically ill patients with acute renal failure. J Am Soc Nephrol 2004;15:2449-2456.

32. Ahlström A, Hynninen M, Tallgren M, et al. Predictive value of interleukins 6, 8 and 10, and low HLA-DR expression in acute renal failure. Clin Nephrol 2004;61:103-110.

33. Han WK, Bonventre JV. Biologic markers for the early detection of acute kidney injury. Curr Opin Crit Care 2004;10:476-482.

34. Parikh CR, Jani A, Melnikov VY, et al. Urinary interleukin-18 is a marker of human acute tubular necrosis. Am J Kidney Dis 2004;43:405-414.

35. Coca SG, Parikh CR. Urinary biomarkers for acute kidney injury: perspectives on translation. Clin J Am Soc Nephrol 2008;3:481-490.
