# Supplementary material for: Expression Profile of Cytokines and Enzymes mRNA in Blood Leukocytes of Dogs with Leptospirosis and Its Associated Pulmonary Hemorrhage Syndrome
Source: PLoS One. 2016 Jan 29;11(1):e0148029. doi: 10.1371/journal.pone.0148029 (PMC4732604; doi:10.1371/journal.pone.0148029)
Supplement: S1 Table — (PDF) [file pone.0148029.s002.pdf]

HC	CKD	AKInL	LEPTO nLAPH	LEPTO LAPH
n=10	n=8	n=14	n=12	n=22
30	9	7	2	17
31	18	16	12	27
34	21	41	15	28
38	25	57	43	33
39	32	71	46	37
40	65	82	51	45
48	69	93	52	47
49	100	95	59	53
50		96	62	55
54		97	70	56
		99	81	58
		101	85	60
		102		66
		103		72
				73
				77
				78
				83
				84
				87
				88
				89

Dog groups: dog ID of the individual dogs and their respective groups (HC, healthy controls; CKD, chronic kidney disease; AKInL, acute kidney injury from other causes than leptospirosis; LEPTO nLAPH, leptospirosis without associated pulmonary hemorrhages; LEPTO LAPH, leptospirosis with associated pulmonary hemorrhages)

Cytokine expression

dog ID	day post admission	UBQ	IL1α	IL1β	IL8	IL10	TNFα	TGFβ	5-LO	INOS
2	1	14.21	14.29	15.94	5.71	7.34	-2.06	15.75	17.79	9.06
2	2	15.02	14.24	16.88	7.55	8.67	-1.25	16.03	18.06	9.72
2	3	15.06	14.1	16.51	6.46	8.31	0.21	16.01	18.45	10.17
7	1	13.81	11.76	12.98	4.28	6.17	11.94	15.82	15.43	4.31
7	2	13.67	12.28	13.77	3.82	7.02	11.9	15.8	15.86	3.62
7	3	13.5	9.68	11.94	2.61	6.38	11.91	14.81	14.95	2.04
9	1	14.32	10.55	13.73	4.66	6.4	12.9	15.32	14.82	5.13
9	2	14.13	11.26	14.39	5.76	6.52	12.55	15.46	15.2	5.78
9	3	14.42	11.51	14.23	7.15	7.11	12.76	15.63	15.53	5.68
12	1	13.77	10.47	13.72	4.25	6.48	10.34	15.15	16.91	5.42
12	2	13.59	10.83	13.71	2.28	6.2	10.37	14.91	16.48	6.04
12	3	13.43	10.36	13.7	3.27	5.86	10.78	15.13	16.31	5.15
15	1	13.95	13	14.63	4.62	6.61	10.75	14.32	17.69	7.36
15	2	14.43	13.72	14.9	6.49	8.42	10.44	14.58	17.18	7.88
15	3	14.37	12.51	14.27	6.41	8.54	10.08	14.8	17.11	7.63
16	1	12.93	13.18	16.27	10.27	3.36	8.24	13.12	15	7.51
16	2	13.48	12.91	16.68	9.58	4.33	8.61	13.75	15.84	6.08
17	1	14.08	14	16.15	7.06	7.47	10.98	15.98	17.87	5.98
17	2	14.39	13.99	17.67	7.7	7.65	10.27	15.91	15.68	8.05
18	1	14.45	13.2	13.65	7.19	8.81	12.59	16.42	16.65	5.05
18	2	14.39	13.98	14.6	8.13	8.59	12.22	16.43	17.29	4.98
18	3	15.38	13.76	15.53	5.98	8.35	13.03	17.19	18.51	7.11
21	1	13.86	11.91	12.52	7.58	6.72	12.08	16.06	17.33	5.47
21	2	13.68	12.49	13.36	7.03	6.55	12.3	15.71	17.44	5.04
25	1	14.36	14.1	16.99	7.89	6.87	11	15.89	18.86	7.78
25	2	14.5	13.69	17.21	9.63	7.34	11.02	16.17	18.22	8.45
25	3	14.56	14.72	17.63	11.2	7.82	11.57	15.89	17.83	9.03
27	1	13.82	10.36	11.65	7	7.58	11.24	14.97	17.22	6.55
27	2	13.7	10.45	12	7.98	7.87	10.92	15.04	16.62	6.4
27	3	13.47	11.22	11.66	8.45	7.52	9.81	14.6	16.01	5.77
28	1	14.16	13.5	15.65	11.6	7.93	10.82	15.88	17.25	4.49
28	2	14.2	13.73	15.98	11.53	8.87	11.13	16.71	18.15	5.72
28	3	14.43	13.85	15.98	11.59	8.41	11.13	15.87	16.7	7.49

30	1	13.78	10.36	14.65	7.18	6.27	11.98	15.43	15.87	4.24
30	2	14.13	11.21	14.61	7.38	6.26	12.1	15.14	15.88	3.89
30	8	13.6	9.84	14.01	7.03	6.02	11.56	15.5	16	4.78
31	1	13.83	9.78	12.12	3.99	6.04	12.26	14.84	15.2	2.81
31	2	13.8	9.71	11.52	5.22	6.28	12.12	15.02	15.03	3.48
31	3	14.09	10	11.74	3.85	6.45	12.25	15.18	15.62	4.18
32	1	14.47	13.49	17.32	8.4	7.94	10.8	15.58	17.98	6.82
32	2	14.33	12.78	15.55	7.53	7.88	10.92	15.82	18.2	7.82
32	3	14.55	12.97	15.95	6.81	8.15	11.57	15.52	17.98	7.04
33	1	13.71	11.5	12.84	-1.8	6.53	9.58	14.6	16.97	5.82
33	2	14.14	12.54	13.8	-1.07	8.16	11.37	15.12	16.32	5.8
33	3	13.59	10.73	12.48	-4.07	7.85	10.83	14.27	15.15	4.33
34	1	13.03	9.51	11.43	6.14	4.54	11.26	13.67	14.38	3.85
34	2	12.86	8.9	11.13	6.04	3.44	10.26	13.16	13.94	4.43
34	3	13	8.73	10.42	5.11	4.21	10.71	13.39	13.4	3.17
37	1	13.68	9.39	11.49	5.06	6.08	10.4	15.01	16.04	5.65
37	2	13.49	9.12	11.12	4.45	6.27	10.35	14.93	15.08	4.63
37	3	13.36	9.08	11.09	5.63	6.81	10.58	15.34	15.71	4.09
38	1	13.11	9.98	11.56	6.8	5.37	10.41	14.26	14.67	4.37
38	2	12.85	8.89	10.77	6.27	4.45	10.31	13.45	14.71	2.83
38	3	13.06	9.42	11.09	6.65	4.71	9.92	14.05	15.18	3.41
39	1	13.57	10.03	12.66	1.75	6.34	11.86	14.98	15.25	4.08
39	2	14.2	10.7	13.79	3.61	6.62	12.88	15.7	12.52	6.96
39	3	13.66	10.48	13.21	4.04	7	12.31	15.2	15.23	3.33
40	1	13.63	11.47	14.64	9.46	6.98	11.56	15.23	16.91	3.82
40	2	14.03	11.72	15.11	8.48	6.37	11.96	15.83	16.94	4.15
40	3	13.74	9.89	13.1	8.92	6.29	11.44	14.94	16.34	4.22
41	1	14.61	13.04	16.37	10.31	7.65	10.21	16.24	18.38	6.25
41	2	14.23	13.5	16.51	9.96	7.25	10.83	15.48	18.39	8.03
41	3	14.42	12.55	16.05	7.71	7.36	11.07	15.9	19.3	6.28
43	1	14.13	10.37	13.82	9.83	9.55	11.28	15.41	18.3	6.15
43	2	14.13	11.04	14.22	10.52	8.76	11.54	15.71	18.58	7.29
43	3	14.07	13.82	16.09	9.61	8.7	11.64	15.24	17.8	5.94
45	1	13.8	12.47	15.25	9.45	7.41	10.06	14.54	16.77	6.2
45	2	13.93	12.53	14.92	4.88	6.5	11.65	14.81	16.99	6.75
45	3	14.22	11.75	14.55	4.14	7.81	11.94	15.83	16.26	5.73

46	1	14.16	12.81	15.94	7.61	6.05	11.34	16.57	18.28	5.99
46	2	14.87	13.27	17.23	7.24	7.66	13.31	16.84	17.41	6.46
46	3	14.88	12.93	17.78	7.11	7.51	13.01	17.42	18.5	7.01
47	1	14.48	12	15.71	8.07	7.54	10.88	15.68	18.05	8.07
47	2	13.89	11.78	15.12	8.16	7.71	10.47	15	17.28	6.87
47	3	14.29	12.99	15.53	10.38	7.68	10.14	15.31	17.63	7.3
48	1	13.17	9.93	12.5	5.24	6.31	11.08	14.74	14.59	4.3
48	2	13.24	10.39	12.5	7.67	6.19	10.64	14.28	14.19	3.49
48	3	13.24	9.91	12.25	7.21	6.05	10.83	14.53	14.53	3.41
49	1	12.81	9.72	12.31	4.78	4.63	11.33	14.56	14.54	2.29
49	2	13.21	10.7	13.22	6.06	4.8	11.87	14.33	14.82	4.1
49	3	12.61	10.06	11.39	6.63	4.29	10.42	13.63	13.88	2.06
50	1	13.13	9.83	11.91	5.74	4.78	10.82	14.15	14.68	3.7
50	2	13.26	10.05	12.62	5.02	5.08	11.43	14.8	15.01	3.51
50	3	12.73	9.31	11.9	6.98	4.6	10.94	13.97	14.31	4.08
51	1	13.45	12.84	16.79	8.96	6.72	10.36	14.29	18.15	8.98
51	2	14.71	14.74	17.65	9.82	8.65	11.85	15.1	18.3	11.01
51	3	14.57	14.38	17.51	9.62	7.95	11.26	14.47	18.3	10.82
52	1	14.38	13.33	13.54	13.71	7.52	10.97	14.54	18.46	8.07
52	2	14.21	12.62	13.57	11.96	7.63	10.46	14.85	18.27	8.2
52	3	13.77	12.78	13.08	5.87	8	10.63	14.55	17.49	6.7
53	1	14.11	14.05	17.23	9.49	8.15	10	14.47	17.6	8.13
53	2	14.56	13.63	16.7	9.27	8.6	9.87	14.61	17.03	8.65
54	1	14.1	12.54	14.33	7.65	5.87	11.71	15.4	16.78	5.5
54	2	14.08	11.8	13.37	2.09	5.82	12.18	15.57	16.7	6.5
54	3	13.91	9.51	12.4	5.24	5.82	11.81	15.41	16.56	6.7
55	1	14.09	12.7	17.08	6.97	6.46	10.01	12.87	17.71	9.74
56	1	14.15	12.19	14.53	7.32	7.67	10.17	14.72	18.43	8.37
56	2	14.45	13.43	15.17	8.61	8.52	10.6	15.33	18.22	8.78
56	3	14.52	13.2	15.56	8.89	5.87	10.58	15.76	18.21	8.75
57	1	14.77	12.42	16.61	9.57	6.86	10.55	16.74	19.72	9.61
57	2	14.64	12.38	17.94	9.17	8.36	11.24	15.63	19	10.53
58	1	13.32	12.55	13.59	0.31	5.11	9.56	13.55	15.79	6.92
58	2	13.75	11.34	13.01	-0.01	5.83	9.97	14.64	14.36	4.85
58	3	13.37	11.05	12.24	0.48	6.07	9.91	14.55	15.48	5.24
59	1	14.46	11.77	13.92	8.22	6.3	11.45	15.16	17.25	7.54

59	2	13.79	11.59	13.82	7.44	6.55	11.11	14.69	16.34	7.72
59	3	13.55	12.23	12.94	7.74	7.68	12.01	14.97	15.53	6.47
60	1	14.11	13.13	14.69	9.64	8.14	9.37	14.62	17.98	9.08
62	1	14.86	13.1	14.72	12.34	7.66	11.09	16.61	18.99	9.94
62	2	14.16	13.26	15.24	10.23	7.88	11.86	15.69	17.1	8.64
62	3	14.21	11.74	14.57	9.44	8.35	11.82	15.6	17.22	10.04
65	1	14.38	11.43	13.15	4.45	7.31	11.09	16.21	16.66	4.33
65	2	14.52	13.5	16.26	7.61	7.45	12.19	16.48	17.17	5.93
65	3	14.41	13.69	16.23	6.12	7.09	13.85	16.39	17.64	5.18
66	1	13.96	13.06	14.11	10.45	7.55	9.34	15.42	17.94	7.17
66	2	14.01	13.2	14.43	11.03	7.48	10.18	15.73	18.19	6.83
66	3	14.15	13.51	16.23	9.66	7.99	11.07	14.94	18.52	6.8
69	1	14.42	14.59	17.65	8.82	7.59	12.47	16.39	18.08	6.81
69	1	14.47	14.68	18.05	9.49	7.25	12.5	16.68	18.32	8.22
69	2	14.46	13.22	17.51	7.91	7.47	11.96	16.19	18.3	9.21
70	1	14.02	11.65	14.3	8.44	7.32	10.01	14.78	16.64	7.24
70	2	14.18	10.93	12.86	8.56	7.59	9.86	14.95	16.14	6.5
70	3	14.51	17.03	16.55	9.95	9.05	13.04	16.03	17.67	6.49
71	1	14.56	14.88	16.59	9.36	6.52	10.97	16.99	19.14	6.62
71	2	15.09	15.54	17.65	10.75	7.3	11.5	17.64	20.08	9.25
71	3	15.26	15.59	18.29	9.98	8.14	11.96	17.75	20.03	8.24
72	1	14.65	13.93	16.53	11.08	7.81	10.65	15.45	18.59	7.6
72	2	14.42	13.44	15.82	10.01	8.45	11.74	16.15	16.91	6.31
72	3	14.52	12.66	15.73	9.6	8.13	11.87	16.43	17.16	5.52
73	1	13.76	11.11	11.6	2.18	7.8	10.87	15.74	15.9	2.27
73	2	13.88	13.05	13.84	1.36	7.56	10.98	16.03	16.81	4.95
73	3	13.2	11.28	13.5	1.59	7.78	11.59	15.44	15.79	1.95
77	1	14.54	12.64	15.06	10.09	9.06	11.55	16.38	18.05	8.79
77	2	14.22	12.51	14.84	7.72	9.05	11.51	16	17.43	6.64
77	3	13.98	11.8	14.23	10.7	8.73	11.13	16.08	16.54	5.91
78	1	14.43	13.68	16.44	8.89	7.22	8.82	14.94	17.16	8.51
81	1	14.03	11.54	13.61	9.41	7.85	10.78	16.31	17.12	6.16
81	2	13.85	12.41	14.1	9.3	7.81	10.88	16.31	17.18	6.34
81	3	13.85	13.43	16.04	4.34	7.98	11.17	16.29	17.47	5.65
82	1	13.38	13.27	14.72	7.68	6.69	11.48	15.96	17.78	4.93
82	2	13.72	14.66	15.47	8.4	7.27	11.79	15.83	17.8	6.7

83	1	13.77	11.45	13.7	8.13	7.95	11.21	15.87	16.03	4.67
83	2	13.85	12.07	14.98	8.62	8.1	11.82	16.21	17.04	5.7
83	3	14.1	13.89	16.15	8.78	8.33	12.18	16.41	17.17	5.79
84	1	14.56	11.29	14.43	10.94	8.52	10.5	14.08	17.24	8.91
84	2	14.2	10.73	14.95	9	8.14	11.01	15	17.29	8.06
84	3	13.93	10.76	14.74	8.37	7.62	10.69	14.44	16.66	7.76
85	1	13.13	10.73	13.17	5.81	5.43	11.59	15.43	15.78	2
85	2	13.75	11.67	13.99	7.25	5.28	11.72	15.45	15.39	4.65
85	3	13.59	11.73	14.28	7.75	7.51	12.54	15.78	16.24	3.39
87	1	14.05	13.16	15.18	9.52	6.56	10.99	14.01	17.04	5.61
87	2	13.79	11.76	13.38	6.13	6.49	11.31	14.7	14.52	4.74
88	1	14.6	12.13	15.49	7.26	6.27	11.7	15.73	18.5	10.5
88	2	14.37	13.99	15.46	10.02	7.53	12.03	15.92	17.86	10.62
88	3	14.32	12.76	15.29	7.9	7.38	12.07	15.53	17.26	9.83
89	1	14.29	14.4	15.21	2.98	7.25	8.89	16.12	18.21	7.5
89	2	14.57	15.06	13.36	4.84	7.73	9.07	16.58	17.81	6.13
89	3	14.44	14.83	16.4		8.13	8.9	17	16.42	6.18
93	1	14.65	15.31	18.54	8.08	6.81	11.34	16.09	18.39	9.03
93	2	14.53	14.18	17.74	8.06	6.97	11.09	15.98	17.72	8.1
93	3	14.02	13.08	16.64	8.51	7.1	11.39	16.07	17.42	7.23
95	1	14.52	12.67	14.91	2.95	7.6	12.7	16.08	12.7	4.96
95	2	14.45	13.03	15.89	2.52	7.62	12.26	16.01	12.26	4.56
95	3	14.09	11.38	14.75	3.54	7.43	11.8	15.69	11.8	4.78
96	1	14.11	10.9	15.72	3.13	8.8	11.09	14.97	11.09	7.04
96	2	13.89	10.54	15.15	2.55	8.07	12.01	14.27	12.01	7.62
96	3	14.02	11.05	13.07	3.9	9.32	12.87	15.46	12.87	8.27
97	1	14.96	14.63	15.98	9.72	7.58	13.37	16.89	13.37	3.95
97	2	14.1	11.98	14.84	7.74	6.41	12.76	16.97	12.76	4.74
97	3	14.37	11.72	13.97	9.78	7.15	12.53	16.82	12.53	4.83
99	1	14.6	11.07	13.13	10.25	7.9	10.96	15.87	10.96	4.97
99	2	14.99	13.05	14.04	9.99	8.4	12.05	16.8	12.05	5.3
99	3	15.07	13.14	15.27	8.71	7.73	12.5	17.2	12.5	6.74
100	1	13.57	12.1	14.41	8.35	5.91	11.55	15.46	11.55	5.82
100	2	14.03	12.73	15.49	8.79	6.98	11.66	15.45	11.66	7.05
100	3	14.19	12.58	15.95	9.24	7.32	11.39	15.2	11.39	8.83
101	1	13.71	11.84	15	-2.22	6.96	9.81	14.61	9.81	8.91

101	2	13.4	11.15	14.74	0.33	7.09	10.43	14.77	10.43	8.66
101	3	13.85	11.86	15.27	-0.62	6.88	9.95	14.28	9.95	8.13
102	1	14.74	11.2	13.33	8.91	8.55	11.62	16.33	11.62	7.07
102	2	14.42	11.94	13.35	8.02	8.33	11.56	15.79	11.56	6.91
102	3	14.89	13.75	14.08	3	8.41	11.28	14.81	11.28	8.12
103	1	14.47	12.18	13.01	9.54	6.75	12.11	15.9	17.05	5.22
103	2	14.22	12.87	14.17	10.63	6.88	11.56	15.86	17.65	7.73
103	3	13.99	11.98	13.87	8.25	6.94	11.42	15.52	16.93	5.82
